# Brain-Stimulation Induced Blindsight: Unconscious Vision or Response Bias?

**DOI:** 10.1371/journal.pone.0082828

**Published:** 2013-12-06

**Authors:** David A. Lloyd, Arman Abrahamyan, Justin A. Harris

**Affiliations:** School of Psychology, University of Sydney, Sydney, New South Wales, Australia; University Medical Center Goettingen, Germany

## Abstract

A dissociation between visual awareness and visual discrimination is referred to as “blindsight”. Blindsight results from loss of function of the primary visual cortex (V1) which can occur due to cerebrovascular accidents (i.e. stroke-related lesions). There are also numerous reports of similar, though reversible, effects on vision induced by transcranial Magnetic Stimulation (TMS) to early visual cortex. These effects point to V1 as the “gate” of visual awareness and have strong implications for understanding the neurological underpinnings of consciousness. It has been argued that evidence for the dissociation between awareness of, and responses to, visual stimuli can be a measurement artifact of the use of a high response criterion under yes-no measures of visual awareness when compared with the criterion free forced-choice responses. This difference between yes-no and forced-choice measures suggests that evidence for a dissociation may actually be normal near-threshold conscious vision. Here we describe three experiments that tested visual performance in normal subjects when their visual awareness was suppressed by applying TMS to the occipital pole. The nature of subjects’ performance whilst undergoing occipital TMS was then verified by use of a psychophysical measure (d') that is independent of response criteria. This showed that there was no genuine dissociation in visual sensitivity measured by yes-no and forced-choice responses. These results highlight that evidence for visual sensitivity in the absence of awareness must be analysed using a bias-free psychophysical measure, such as d', In order to confirm whether or not visual performance is truly unconscious.

## Introduction

Loss of function of the primary visual cortex (V1), which may result from cerebrovascular accidents (i.e. stroke-related lesions), causes a scotoma (an area of blindness) in the corresponding region of the visual field [1]. One of the most intriguing observations in neuropsychology over the last several decades has been evidence from patients with V1 damage who show residual visual function within their scotomata. This phenomenon, known as blindsight, suggests unconscious visual processing and points to V1 as an important neural substrate of visual awareness. Blindsight has been found to occur in a range of different visual tasks, including those involving: detection of visual stimuli [2-5]; localization of targets using saccadic eye movements [6,7]; discrimination of letters, colours, and motion [8,9]; and even recognition of different facial expressions , known as “affective blindsight” [10]. In recent years there has been a number of studies reporting similar effects on visual performance in neurologically normal subjects receiving TMS to early visual cortex [11-14]. However, some authors argue that visual behaviour may take on the appearance of blindsight due to response bias [15,16].

Experimentally defined, blindsight is above-chance performance on forced-choice (FC) visual tasks in the absence of conscious visual awareness. Visual awareness is typically measured using a yes-no (YN) procedure, in which a “yes” response indicates awareness of a visual stimulus and “no” a lack thereof. FC tests, on the other hand, require subjects to make a judgment about a specific property of the stimulus by choosing from a limited set of alternative responses. For example, the subject might have to report whether the stimulus was horizontal or vertical, green or red, or whether it occurred in the first or second interval of a trial. In blindsight, FC accuracy exceeds chance-level on trials when subjects are reportedly not aware of the visual stimulus (they reported “no” to having seen the stimulus). Blindsight performance can reach levels of up to 85% discrimination accuracy for reportedly unseen visual stimuli [9], although these levels are generally around 60-70% [5,17]. Nevertheless, it is possible for a dissociation between YN and FC performance to occur solely due to the use of different response criteria for the YN and FC responses.

**Figure 1:**
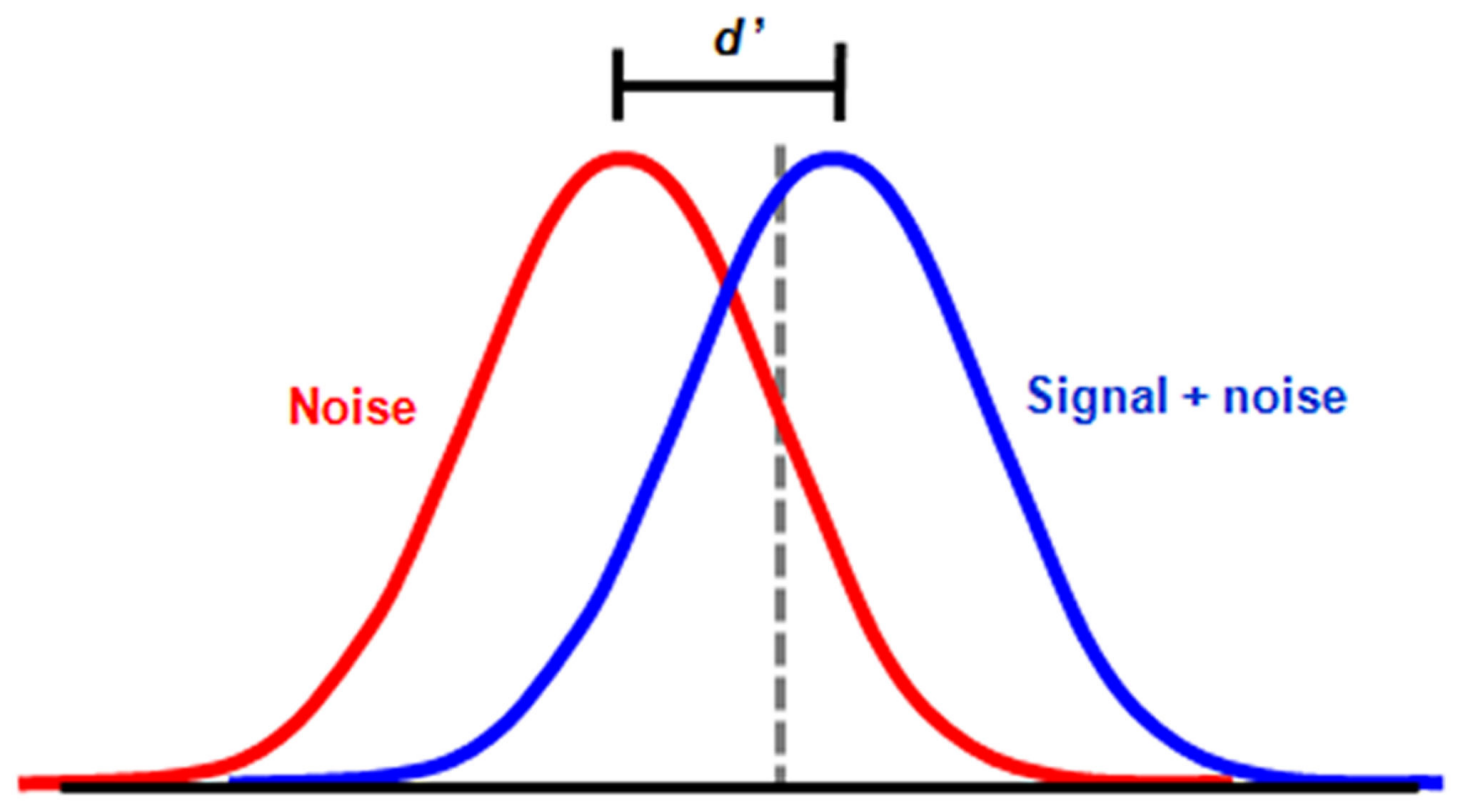
Signal Detection Theory. Normal distributions representing the internal neural activity associated with noise (no stimulus) and signal-plus-noise (stimulus). True sensitivity to the stimulus is denoted d'. The response criterion is represented by the dotted line and reflects the subjective level at which noise is distinguished from signal. In this example, the subject uses a high criterion and is more inclined to say “no” to stimulus presentation than to say “yes”.

In Signal Detection Theory (SDT) [18], the response criterion (also known as bias) used in a YN task is the subjective cut-off point that the observer uses to differentiate sensory noise from that of signal (Figure 1). Above the criterion the observer reports "yes" and below it reports "no". It is almost inevitable that some portion of the stimulus distribution will fall below the observer’s criterion. This causes an observer to disregard residual visual signals when responding "no" (reporting that he/she is unaware of a stimulus). In turn, these residual visual signals are able to produce visual behaviour that is suggestive of unconscious vision, such as in blindsight. On this basis, it has been argued that evidence for blindsight may be accounted for by residual vision associated with the YN decision criterion [15,16]. A relatively high criterion, one which leads the observer to reject a larger proportion of stimulus presentations than were actually not visible, may be adopted by patients with lesion-induced visual defects due to the fact that healthy vision is retained in the remainder of the visual field. This may cause highly degraded visual percepts that occur within scotomata to be rejected relative to intact visual percepts experienced in unaffected parts of the visual field. Similarly, in subjects with TMS-induced visual deficits, it is possible that the participant’s response criterion is systematically affected (raised) due to the presence of phosphenes, which are illusory flashes of light caused by electrical stimulation of visual cortex. Subjects may adopt a high criterion in order to decrease the likelihood of false alarms, in which they respond “yes” to a phosphene on a no-stimulus trial.

In SDT, true (bias free) sensitivity to a stimulus can be measured as d', which takes into account the rate of both correct responses (hits, correct rejections) and incorrect responses (misses, false alarms). For example, an observer’s bias to respond “yes” to seeing the stimulus, which leads to a high hit rate, is accounted for by the corresponding increase in false alarms. d' therefore provides a means by which to measure a person’s sensitivity to seeing a visual stimulus independently of their response bias.

There is need for more systematic use of d' in studies of blindsight, given evidence which suggests bias may contribute to extant cases of the condition. Overgaard, Fehl, Mouridsen, Bergholt, and Cleeremans [19], for example, showed that a blindsight subject, GR, experienced conscious residual vision in the defective visual region associated with her blindsight. This was demonstrated by using an incremental, rather than dichotomous, scale of visual awareness, whereby the lowest level on the scale, labelled "nothing seen", was not associated with above-chance FC accuracy. It was only with higher levels on the scale, which are associated with weak through to relatively strong visual experiences, that such performance occurred. The same was also found in a previous study involving neurologically healthy populations who underwent TMS to occipital cortex [20]. By contrast, Azzopardi and Cowey [2,3] found that another blindsight patient, GY, did differ in terms of his visual sensitivity (d’) between the YN and FC tasks. Similarly [13], found that d’ was greater when normal subjects, after receiving TMS to the occipital cortex, reported the emotion of animated faces compared to when localizing these stimuli, a phenomenon they called TMS-induced “affective” blindsight.

It is important that these discrepancies in the data are resolved, based on the fact that there has been an increase in research interest in this area over the last decade. This interest has been most apparent in regard to studies using TMS to induce an apparently similar visual phenomenon in neurologically normal subjects. Ro et al. [14], for example, found that healthy subjects were able to localise visual stimuli using saccadic eye movements, despite reporting that they did not see these stimuli following TMS stimulation of occipital cortex. Similarly, Boyer et al. [11] found evidence for unconscious visual processing of colour and orientation in the TMS-induced absence of visual awareness. In addition, there has been even more recent TMS-based evidence which suggests that visually-guided motion remains intact without visual awareness, a phenomenon which Christensen et al. [12] have called “action blindsight”. These highly interesting findings hold considerable potential for the greater understanding of human consciousness and its relation to other cognitive processes. Nevertheless, as with evidence for blindsight in neurological patients, it is necessary to validate the authenticity of reportedly unconscious visual accuracy by using bias-free measures of visual performance. 

The aim of the present study was to investigate the issue of response bias during reportedly unconscious vision in subjects receiving TMS to occipital cortex. This was achieved by comparing visual sensitivity, measured as d', between the YN and FC tasks in a bias-free manner. TMS was used to inhibit occipital functioning in neurologically healthy subjects and hence reduce their visual awareness of the test stimuli. Three separate experiments were conducted, each of which used different response methodology to seek convergent evidence for the nature of reportedly unconscious visual behaviour. In all three experiments, response patterns were indicative of what previous studies have called TMS-induced blindsight, but in all instances d' analysis confirmed that this behaviour was a function of response bias rather than a genuine dissociation in visual sensitivity.

## Experiment 1

 The purpose of Experiment 1 was to replicate the procedure used by previous studies of TMS-induced unconscious vision [11,14] and in addition employ SDT in the analysis of evidence of unconscious vision. On each trial, a visual stimulus (a bar orientated 45° to either the left or right) was presented, or no stimulus was presented, followed after 100ms by a TMS pulse to occipital cortex or a control location (Cz). An asynchrony of 100ms between stimulus-onset and TMS was chosen based on previous studies showing that this temporal delay results in significantly reduced visual awareness [11,13,21]. After presentation of the visual stimulus and TMS, subjects made a yes-no (YN) response about their awareness of the stimulus followed by a two-alternative (left or right) forced-choice judgment (FC) about the orientation of the stimulus. During the YN task, subjects were instructed to report “yes” if they saw anything at the stimulus location during a trial. Data from the YN and FC responses were used to determine whether TMS-induced disruption of occipital cortex led to above-chance FC accuracy on trials where subjects reported “no” to having seen the stimulus. Based on previous studies, it was hypothesised that such evidence for unconscious visual performance would be demonstrated during TMS to occipital cortex, but not during TMS to Cz. Sensitivity (d') for the YN and FC tasks was calculated to verify whether reportedly unconscious visual behaviour was due to genuine differences in visual sensitivity, or an artifact arising from the use of a response criterion in the YN task.

In addition, subjects rated their confidence in the correctness of both their YN and FC judgments immediately after making each of these responses. That is, on each trial, subjects gave their YN response and immediately made a confidence rating of that judgment, then they gave the FC response and immediately made a confidence rating about that response. Confidence ratings were used to construct Receiver Operating Characteristic (ROC) curves, which give an indication of d' by plotting hit rate against false-alarm (FA) rate. Given that confidence ratings represent different levels of a decision criterion, ROC curves may give a different reading of visual sensitivity to actual YN and FC responses. Furthermore, the confidence scale, by virtue of having more than 2 levels on which to make a response, is more ‘exhaustive’ than the YN measure and therefore more likely to detect evidence of residual vision than the YN measure [22].

### Methods

#### Ethics statement

This research (“The Effects of Transcranial Magnetic Stimulation (TMS) on Visual Perception”) was approved by the Human Research Ethics Committee at the University of Sydney. Approval number: 11-2006/9632.

#### Subjects and apparatus

**Subjects**: Ten volunteers (3 female; mean age 29.9; age range, 23 to 50 years), including the three authors, participated in this experiment. The subjects were neurologically healthy, and had normal or corrected-to-normal vision. All subjects gave written, informed consent and completed a TMS safety-screening questionnaire prior to completing the experiment.

**Visual stimulus**: The visual stimulus was a grey bar (width = 1°, height = 2.5°), oriented 45° to the left or right of vertical, multiplied by a symmetrical two-dimensional Gaussian contrast envelope to eliminate sharp edges. The stimulus was presented within a black circular ring that subtended a 5° visual angle against a uniform grey background of 77cd/m^2^. The stimulus contrast was set to the subject’s individual contrast threshold for detection, as described below.

**Equipment**: Visual stimuli were presented on a 19² cathode ray tube monitor (P992, BenQ) operating at a 75Hz refresh rate and 1024x768 screen resolution. A Bits++ digital video processor (CRS, Cambridge, UK) was used to increase the contrast range of the PC’s graphics card from 8 bits (the default) to 14 bits. This extended the available luminance levels from 256 to 8192. Gamma correction was also applied using an OptiCAL photometer (CRS, Cambridge, UK) and a Matlab script to ensure a linear luminance profile. The subject’s head was supported by chin and forehead rests at a viewing distance of 57cm from the monitor. Stimulus presentation and synchronous triggering of the TMS pulse were programmed in Matlab (Mathworks), assisted by Psychtoolbox [23]. TMS was delivered using a Magstim Rapid^2^ system (Whitland, UK) with a 70mm figure-eight coil held by an adjustable articulated arm (Manfrotto, Italy). To ensure consistent positioning of the coil throughout the experiment, head movements were tracked with a real-time 3D neuro-navigation system (Brainsight, Rogue Research, Canada). 

### Procedure

#### TMS protocol

Subjects were seated in a dimly lit room and allowed to dark adapt for approximately 5min. The TMS coil was positioned with the handle oriented horizontally, pointing to the left of the subject and tangential to the scalp. The coil was initially placed over the back of the subject’s head, with the centre over an area 3-5cm above the inion. Single pulses were delivered at intensities that reached maximum stimulator output, while the coil was moved in steps of 0.5-1cm. The TMS position on the back of the head that evoked the strongest phosphenes was marked as a “hotspot”. The coil was fixed in this position using the articulated arm, and the neuro-navigation system tracked the coil’s position relative to the subject’s head, ensuring that it remained within 2mm of the “hotspot” throughout the experiment. Subjects indicated the location of their phosphenes using the mouse to move a circle (6° x 6.5° of visual angle) on the monitor while they maintained fixation at a marker at the centre of the monitor. During this procedure the experimenter delivered additional TMS pulses, as requested by the subject, to help the subject match the position of the circle with the location of the phosphene. 

The control site for TMS stimulation was Cz. This is a non-visual region located at the intersection of the midline (from nasion to inion) and the inter-aural line. The stimulation of Cz allowed the reproduction of non-specific TMS effects, such as the auditory and tactile effects of a TMS pulse, but without impacting on visual cortex in posterior regions of the brain. In the Cz condition, the coil was placed horizontally on the top of the subject’s head, with the handle pointing backwards, and the centre of the coil over Cz.

#### Phosphene threshold measurement

Phosphenes served as a method for titrating the strength of the TMS pulse relative to the visuo-cortical threshold of the individual subject. The locations where the subject sees phosphenes also indicates the receptive field of the stimulated neurons, within which the visual stimulus can be positioned.

To measure the phosphene threshold, the subject was asked to close his or her eyes while maintaining fixation on the remembered location at the centre of the screen. The threshold procedure used a Matlab toolbox combined with a Bayesian adaptive staircase [24], a method developed by Abrahamyan et al. [25]. On each of the 30 trials within a staircase, a single TMS pulse of variable intensity was delivered, after which the subject reported by key press whether or not they saw a phosphene. The phosphene threshold was calculated as the average of two staircases and corresponded to the stimulation intensity (as percentage of maximum TMS output) that evoked phosphenes on 60% of trials. Across the three experiments, phosphene thresholds ranged from 50 - 80% of maximum TMS output.

Phosphenes were located in the lower right quadrant of the visual field for 6 subjects (CW, CM, NM, DL, SL, JH). For subject AL the phosphene was located in the right visual field along the horizontal axis and thus extending into the upper right quadrant of the visual field. For the 3 remaining subjects (WC, MK, AA), phosphenes were elicited in the lower left quadrant of the visual field. During the experiment, the visual stimulus was always positioned within the area that the subject had previously identified to be the location of the phosphene. 

#### Contrast threshold measurement

The contrast threshold for the visual stimulus was measured using a dual-staircase, two-alternative forced-choice task (FC). The beginning of each trial was marked by a central fixation cross (0.7° of visual angle) remaining on the screen for 346 ms (26 frames). As a cue to the stimulus, the fixation cross then changed into a square (0.2° of visual angle). After another 346 ms, a short beep was presented as an auditory cue, while the fixation square remained on the screen for a further 346 ms before the visual stimulus appeared on the screen. The stimulus was presented for 40ms (3 frames) within a black circular ring located in the previously established phosphene area. Subjects reported by key press whether the stimulus was oriented to the left (using the > key) or right (using the ? key). The stimulus contrast was varied across trials according to a Bayesian adaptive staircase [24]. After the 60-trial block, in which two 30-trial staircases were run in an interleaved fashion, an estimate of contrast threshold was obtained that corresponded to an accuracy level of 80.3%. Two contrast threshold procedures were run for each subject and the average of these was the contrast estimate that was used.

During the contrast threshold procedure and the actual experiment (described below), visual stimuli were predominantly located in peripheral regions of the lower-half of the visual field, as this is where phosphenes regularly occurred. To ensure that peripheral stimuli did not cause deviations in central fixation during the experiment, a central fixation marker remained present on the computer monitor throughout each trial. Subjects were instructed to fixate the central marker throughout the experiment. It is recognised that saccadic eye movements to phosphenes may have occurred, as Schiller and Tehovnik [26] have reported, however this would not have affected subjects performance since the TMS pulse occurred a considerable amount of time (i.e. 60ms) after stimulus offset. 

#### Visual detection task

During the visual detection task, presentation conditions for the visual stimulus were identical to those used in the contrast threshold procedure, except that the contrast of the stimulus was fixed at the detection threshold level of the individual subject. Furthermore, stimulus-present trials (66.6%) were randomly intermixed with blank (i.e. stimulus-absent) trials (33.3%). Subjects were informed that some trials would be blank, but were not told of the proportion of stimulus to blank trials to avoid influencing their response criteria.

Each subject completed the detection task under three different conditions: (1) a TMS pulse was delivered to the occipital cortex “hotspot”; (2) a TMS pulse was delivered to Cz; (3) no TMS was administered. In conditions 1 and 2, TMS was administered at levels corresponding to 120% of the phosphene threshold for each subject. Two control conditions, Cz and no TMS, were used to test whether the mere presence of the TMS, and other extraneous TMS factors, affected visual performance. The three conditions were randomly intermixed in different 30-trial blocks and all subjects completed at least 5 blocks (150 trials) per condition.

At the end of each trial, subjects answered four questions. The first question asked whether or not they had seen the stimulus, to which they verbally responded “yes” or “no”. Subjects then had to rate how confident they were in the correctness of their YN decision by pressing one of four keys with their left hand, which corresponded to: ‘very confident’ (z), ‘somewhat confident’ (x), ‘a little confident’ (c), and ‘not at all confident’ (d). The third question asked subjects to indicate the orientation of the stimulus by pressing one of two keys (> and ?) with their right hand. Importantly, if they had not seen the stimulus the subjects were instructed to ‘guess as best they could by making use of any sensory information they had available’. Subjects then rated their confidence in the correctness of their orientation judgment using the same scale and response keys as in step two.

### Analysis.

#### Validation of Cz

To establish that Cz was a valid control for comparison with stimulation of occipital cortex, we sought to test if the extraneous TMS factors acting in the Cz condition led to significantly different visual performance to that found under no TMS stimulation. This was examined using a paired-samples t-test, comparing the effects of condition (Cz and no TMS) on sensitivity (d') in the YN task.

#### Measurement of TMS-induced effects on visual performance

Evidence of unconscious visual performance in both the occipital cortex and Cz conditions was sought by calculation of FC accuracy (percentage of correct orientation judgments) on stimulus-present trials where the subject responded “no” to seeing the stimulus.

#### Validation of reportedly unconscious vision during TMS

To determine whether reportedly unconscious vision induced by occipital TMS is due to greater sensitivity under FC response conditions, a 2x2 repeated measures ANOVA was performed comparing the effects of condition (occipital cortex and Cz) and task (YN and FC) on sensitivity (d'). The main effect of condition (occipital cortex vs Cz) on visual sensitivity across the YN and FC tasks established whether TMS to occipital cortex caused significant impairments to visual awareness. The critical test was that of the interaction between condition (occipital cortex vs Cz) and task (YN vs FC). The interaction tested whether or not FC sensitivity was greater than YN sensitivity under TMS stimulation of occipital cortex, and whether or not this difference was significantly greater than the corresponding comparison in the Cz condition. Demonstration of such an interaction would indicate that TMS-induced loss of function of occipital cortex has a specific effect on neural functioning leading to above-chance FC accuracy in the absence of conscious visual awareness.

#### Receiver Operating Characteristic (ROC) curves

In the final part of the analysis, ROC sensitivity curves were constructed from confidence ratings in YN and FC responses. As the ROCs were based on confidence ratings for YN and FC responses, rather than the responses per se, the curves provided a complementary picture of sensitivity to that found in the previous stage of the analysis. ROCs were drawn from confidence ratings by re-categorising YN and FC responses according to these ratings. For the FC task, the responses were first re-classified such that presentations of the left bar represented “target present” trials and presentations of the right bar represented “target absent” trials to allow 2AFC data to be treated in the same way as YN data. The re-categorisation of responses based on confidence ratings generated an alternative hit-rate and false-alarm rate for all but the end point on the confidence scale. Since ROCs were based on confidence ratings for polar responses (e.g. Yes and No), the confidence scale for “no” responses was reversed. This effectively doubled the number of the points in the confidence scale from 4 to 8, allowing the construction of ROCs based on 7 pairs of hit and false-alarm rates.

The ROCs of interest in this analysis were those based on confidence ratings for “no” responses in the YN task on trials where the stimulus was presented (YN ROC for misses) and those fitted to confidence ratings for FC judgments on these same “no” trials (FC ROC for misses), as these represent the responses of interest in TMS-induced unconscious vision. These ROCs indicate whether or not subjects had residual sensitivity to the visual stimulus on trials where they reported not seeing it. The ROC analysis calculated the level of sensitivity evident in each of the ROC curves for individual subjects, as well as the mean difference in sensitivity between the two ROCs. The mean difference in sensitivity between the ROCs was used to determine whether FC performance was surmounted by potentially “unconscious” residual vision.

### Results

#### Cz vs no TMS

Sensitivity in the YN task was not significantly different, on average, between the Cz condition (d' = 1.55, SE = .17) and no TMS condition (d' = 1.39, SE = .13), *t*_8_ = - 1.00, *p* = .35, nor was sensitivity in the FC task significantly different between these two conditions (d'_Cz_ = 1.44, SE =.21; d'_no TMS_ = 1.17, SE = .24), *t*_8_ = - 1.71, *p* = .28. This finding indicates that the mere presence of the TMS, and other extraneous TMS factors, did not significantly impact upon visual performance.

#### Occipital cortex vs Cz

**Figure 2:**
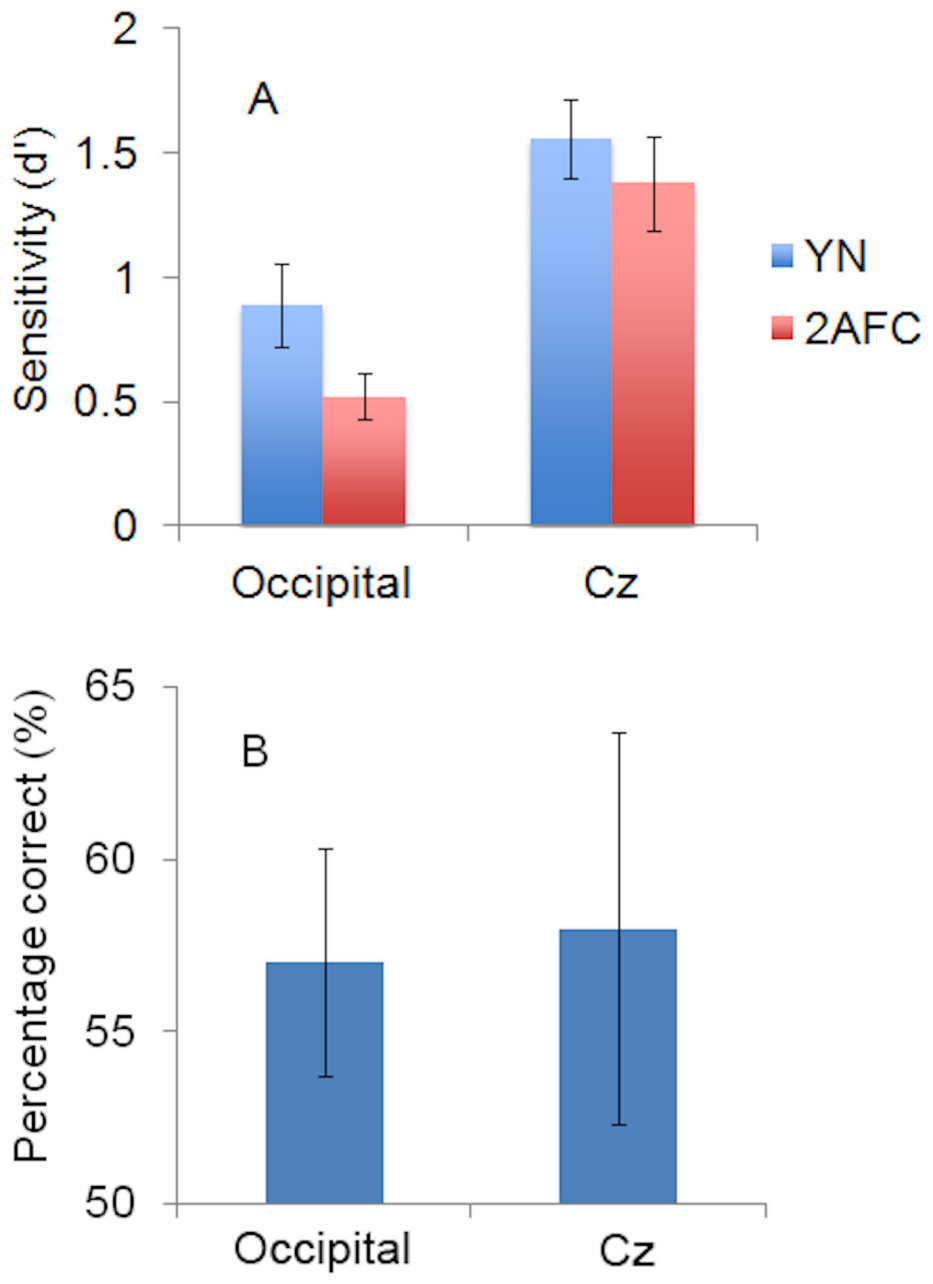
Evidence for TMS-induced Unconscious Vision. **A**. Visual sensitivity under Yes-No (YN) and Forced-Choice (FC) response conditions during TMS stimulation of Occipital Cortex and Cz. **B**. Percentage of correct orientation judgments on miss trials during TMS stimulation of Occipital Cortex and Cz. Vertical bars show the standard error of the mean.

The 2x2 repeated measures ANOVA indicated a significant main effect of condition (occipital cortex vs Cz), such that visual sensitivity (d') was significantly lower during stimulation of occipital cortex (d' = .71, SE = .13) than during stimulation at Cz (d' = 1.47, SE = .18), *F*_1, 9_ = 14.72, *p* = .004. This confirms that TMS to occipital cortex had a significant detrimental effect on visual awareness. The main effect of test (YN vs FC) was not significant (d'_YN_ = 1.23, SE = .16, d'_FC_ = .95, SE = .14, F_1, 9_ = 2.259, *p* = .167), indicating that visual performance was consistent between the YN and FC tasks independently of other factors. During occipital cortex stimulation, sensitivity in the FC task was less than that in the YN task (mean difference = .37, SE = .20), a finding which is inconsistent with the notion of TMS-induced unconscious vision. Furthermore, this difference was not significantly different to the corresponding comparison in the Cz condition (mean difference = .18, SE = .20), *F*_(1, 9)_ = .90, *p* = .368. The absence of interaction between the site of TMS stimulation and visual sensitivity in the YN and FC tasks, illustrated in Figure 2A, points out that the application of TMS to occipital cortex did not cause a change in visual functioning that could be interpreted as unconscious vision. Nevertheless, a dissociation was found based on conventional indices (i.e. percentage of correct responses), which are not independent of response bias. As shown in Figure 2B, mean percent correct on miss trials was 57% during occipital cortex stimulation (SE = .03) and 58% (SE = .06) during Cz stimulation. Evidence of reportedly unconscious vision did not differ significantly between the occipital cortex and Cz conditions (M diff = .07, SE = .07, t_9_ = .11, *p* = .92). FC accuracy in the reported absence of visual awareness was not significantly above-chance (*t* test-value = 50%) in the Cz condition (*t*_9_ = 1.40, *p* = .20), but was approaching significance in the occipital cortex condition (*t*_9_ = 2.20, *p* = .056).

#### ROC results

Two types of ROC sensitivity curves were constructed: (1) ROCs based on confidence ratings for “no” responses on stimulus-present trials in the YN task (YN ROC for misses); and (2) ROCs fitted to confidence ratings for FC judgments on these same “no” trials (FC ROC for misses). The ratings reflected subjects’ confidence in the correctness of their responses.

**Figure 3:**
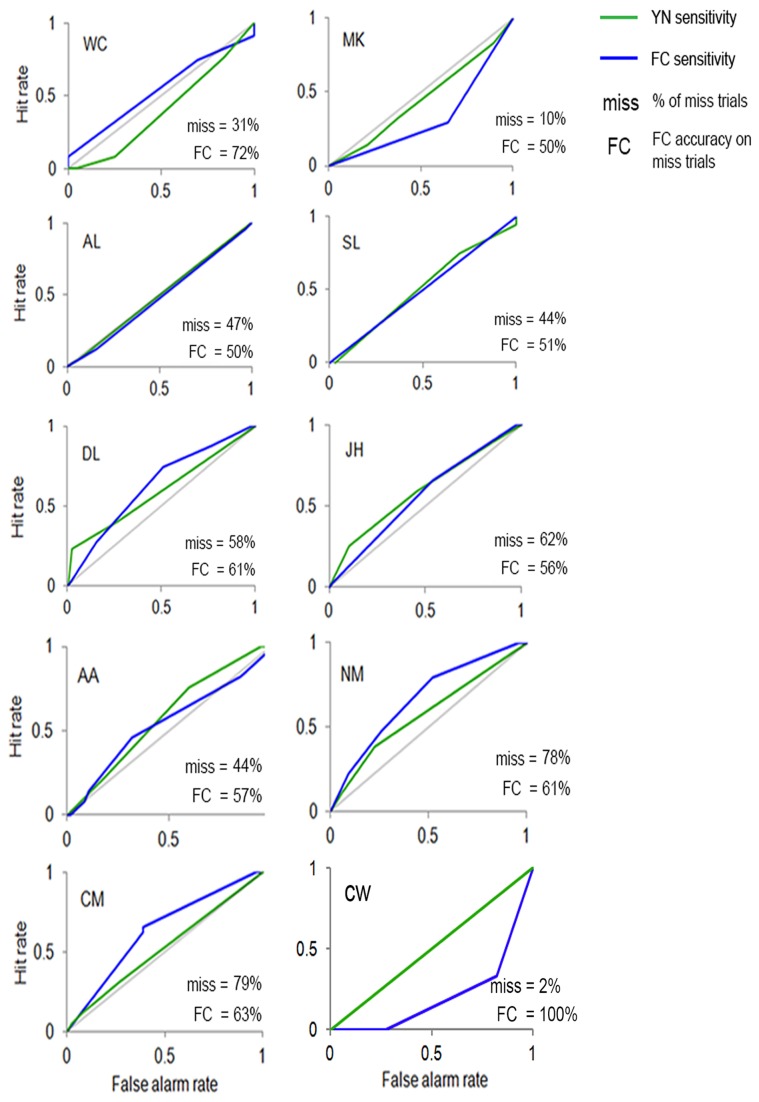
ROC curves. ROCs representing visual sensitivity in the absence of visual awareness under TMS stimulation of Occipital Cortex. The green lines represent ROCs fitted to confidence ratings for “no” responses in the Yes-No task, which refers to when subjects said they did not see the visual stimulus. The ROCs in blue are based on confidence ratings for forced-choice judgments that subjects made after reporting “no” to seeing the stimulus.

During stimulation of occipital cortex (displayed for each individual subject in Figure 3, there was no sensitivity evident in the YN ROCs for misses or the FC ROCs for misses of four subjects (WC, MK, AL, SL). In four other subjects (DL, JH, AA, NM) sensitivity was evident in their YN ROCs for misses and FC ROCs for misses. In only one subject, CM, there was evidence of sensitivity evident in the FC ROC for misses, but no sensitivity evident in the YN ROC for misses. In subject CW, there were too few miss trials (2%) to be able to accurately assess unconscious visual performance. Across all subjects (excluding CW), there was no significant difference (*t*_8_ = - 1.14, SE = .02, *p* = .29) in sensitivity (equal to the area under the ROC curve) based on the YN ROCs for misses and their FC ROCs for misses.

**Figure 4:**
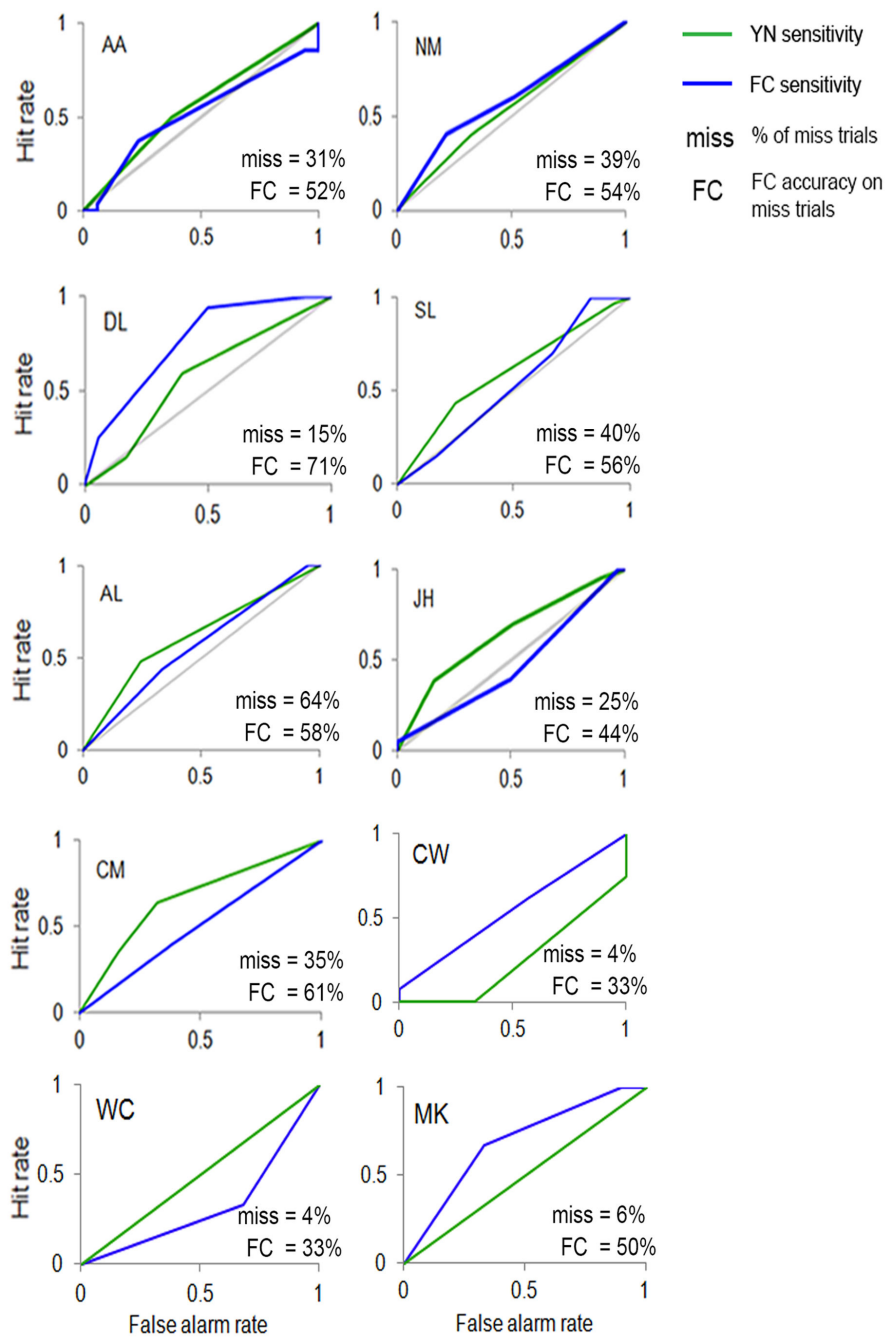
ROC curves. ROCs representing visual sensitivity in the absence of visual awareness after TMS stimulation of Cz. The green lines show ROCs fitted to confidence ratings for “no” responses in the Yes-No task, which refers to when the subject said they did not see the visual stimulus. The ROCs in blue were based on forced-choice judgments that were made after subjects reported “no” to seeing the visual stimulus.

During stimulation of Cz (displayed for each individual subject in Figure 4, sensitivity was evident in both the YN ROCs for misses and FC ROCs for misses of five subjects (AA, NM, DL, SL, AL). In two other subjects (JH, CM), no sensitivity was evident in their FC ROCs for misses, whilst high levels of sensitivity were indicated by their YN ROCs for misses. In three subjects (CW, WC, MK), miss trials percentages were too low to be able to accurately assess unconscious visual performance. The mean sensitivity between the YN ROCs for misses and FC ROCs for misses under Cz stimulation was not significantly different (*t*_7_ = - .20, SE = .04, *p* = .85).

### Discussion

In Experiment 1, TMS was applied to the visual cortex, inducing a temporary disruption in the corresponding part of the visual field. A small bar tilted to the left or right was presented within the TMS-affected region and subjects had to report whether they were aware of the bar (a Yes-No question) and what they thought its orientation was (a forced-choice question). This revealed that during TMS to occipital cortex subjects were able to judge the orientation of the bar at above-chance levels on trials where they reported no awareness of the stimulus. A typical explanation for this finding is that the temporary inhibition of occipital cortex activity triggered unconscious visual processing, which led to greater visual sensitivity under forced-choice (FC) conditions than under those of the Yes-No (YN) task [11,13,14]. However, two findings argue strongly against this conclusion. First, FC performance on missed trials was equivalent in both occipital cortex and Cz conditions, showing that the evidence for TMS-induced unconscious vision, such as it is, was not specific to stimulation of visual cortex. Second, when we used an unbiased measure of sensitivity (d'), we found there was no difference between FC and YN performance during TMS of occipital cortex. Hence evidence for unconscious vision was disconfirmed. Rather, the findings suggest that visual performance reflected low-level, residual vision, which arose as an artifact of the use of a response criterion during the YN assessment of awareness [15].

To better understand the nature of subjects’ visual experiences during the experiment, we also constructed ROC sensitivity curves based on the confidence ratings that subjects made for their YN and FC judgments. Specifically, two different ROCs were analysed: (1) those based on confidence ratings for “no” responses on stimulus-present trials during the YN task (YN ROC for misses); (2) those fitted to confidence ratings for FC judgments made on these same “no” trials (FC ROC for misses). Only miss trials were included in the analysis to account for the fact that TMS suppression of visual awareness does not occur on every trial.

There were two predominant relationships between the ROCs and the extent to which subjects demonstrated TMS-induced unconscious vision.

#### no TMS-induced unconscious vision / no residual visual sensitivity

In four subjects (WC, MK, AL, SL), no sensitivity to the visual stimulus was evident in either the YN ROC for misses or FC ROC for misses (i.e. the ROCs did not show upward deviation from the positive diagonal) during stimulation of occipital cortex. This suggests that these particular subjects had no residual visual sensitivity on trials where they responded “no” to seeing the visual stimulus and were truly guessing the FC response. In accordance with these data, the present subjects were not above-chance when making judgments of the orientation of the unseen visual stimulus.

#### TMS-induced unconscious vision / residual visual sensitivity

In four other subjects (DL, JH, AA, NM), sensitivity was evident in the YN ROC for misses and FC ROC for misses during stimulation of occipital cortex. This points to the presence of residual visual signals in the sensory system on trials where subjects were reportedly unaware of the visual stimulus. This was a consequence of the fact that “yes” and “no” judgments were made about sometimes highly ambiguous visual experiences. These subjects reported “no” to seeing the visual stimulus, even when visual sensitivity was above-zero. Hence the FC response does not appear to have been completely unconscious. This may explain why these observers, in contrast to those previously discussed, performed above-chance when guessing the orientation of the reportedly unseen visual stimulus. What is more, visual performance on the FC task was not surmounted by additional “unconscious” visual information, given sensitivity between the YN for misses and FC ROC for misses did not differ significantly under occipital cortex stimulation.

During TMS stimulation of Cz, there was also evidence of residual visual sensitivity on trials where subjects were reportedly unaware of the visual stimulus. For seven subjects (AA, NM, DL, SL, AL, JH, CM) in this condition, sensitivity was evident in the YN ROC for misses. As previously mentioned, this indicates that when these subjects reported “no” to seeing the visual stimulus this did not reflect a state of zero visual sensitivity. As before, this implies that the FC response in the reported absence of awareness was facilitated by residual visual signals in the sensory system. It was also found that, as in the occipital condition, these conscious visual signals were not augmented by potentially “unconscious” visual information based on the finding that sensitivity levels between the YN ROC for misses and the FC ROC for misses did not significantly differ. Thus conscious low-level vision may also explain the reportedly unconscious vision found during undisrupted occipital cortex function.

### Conclusion

The findings from the ROCs support the conclusion made in the previous part of Experiment 1, which is that residual visual signals produced evidence of TMS-induced unconscious vision due to bias in the YN judgment. ROCs indicated the presence of residual vision in both the occipital and Cz conditions, which explains why we found evidence of TMS-induced unconscious vision irrespective of the site of TMS stimulation.

## Experiment 2

Experiment 2 used the same procedure as that used in Experiment 1 but with two exceptions. The first was that subjects did not rate their confidence in their YN and FC responses as they had in the first experiment. This reduced the response procedure to two steps only, decreasing the time interval between presentation of the stimulus and the subject’s FC response to its orientation. We reasoned that a shorter response procedure might more effectively capture unconscious neural signals. Confidence ratings were also omitted from Experiment 2 in order to simplify the response demands on the subjects, allowing us to test an experimentally inexperienced undergraduate cohort.

The second difference between Experiment 1 and 2 was that subjects in Experiment 2 were asked to respond “yes” or “no” about their awareness of the *orientation* of the stimulus, rather than their awareness of *anything at all*, as they had been asked in Experiment 1. The rationale for this change was based on Boyer et al. [11], who argued that asking subjects about their awareness of *anything at all* restricts TMS-induced unconscious vision to what in blindsight terms is called a “type 1” condition, whereby subjects have absolutely no awareness of visual stimuli presented within their defective visual regions. Boyer et al. [11] suggest that asking subjects about their awareness of the *orientation* of visual stimuli allows for “type 2” blindsight, in which subjects do have conscious, but not explicitly visual, sensations of reportedly unseen visual stimuli. Previous research suggests, however, that “type 2” blindsight may once again be a misinterpretation of conscious, near-threshold vision [19,27,28]. Thus, it was hypothesised for this experiment that FC accuracy in the reported absence of visual awareness would be above-chance under TMS to occipital cortex, but would not reflect true differences in visual sensitivity (d') between the YN and FC tasks. Rather, it was predicted that high response criteria in the YN decision would have made it appear as though subjects were able to discriminate the orientation of visual stimuli without actual awareness.

### Methods

#### Subjects

Ten (four female; mean age 19; age range, 18 to 35 years) first-year psychology undergraduate students participated in this Experiment in exchange for course credit. They were neurologically healthy and had normal or corrected-to-normal vision. All subjects gave written informed consent and completed a TMS safety-screening questionnaire prior to completing the experiment, which was approved by the Human Research Ethics Committee at the University of Sydney. All other aspects of the methodology in Experiment 2, aside from those mentioned in the Introduction, were identical to Experiment 1.

#### Phosphene location

 Phosphenes (and thus visual stimuli) were located in the lower right quadrant of the visual field for 6 subjects and in the lower left quadrant of the visual field for 4 other subjects. Phosphenes did not occur exclusively in the upper half of the visual field, but did extend into this area from the lower right quadrant for 1 subject. 

### Results

#### Cz vs no TMS

Sensitivity in the YN task was not significantly different, on average, between the Cz condition (d' = 2.59, SE = .32) and no TMS condition (d' = 2.49, SE = .28), t_9_ = -.33, *p* = .75, nor was sensitivity in the FC task significantly different between these two conditions, (d'_Cz_ = 2.19, SE =.15; d'_no TMS_ = 2.13, SE = .21), *t*_9_ = - .50, *p* = .63. This finding reiterates that extraneous TMS factors did not have a significant impact on visual performance, and confirms that Cz stimulation was a valid control against which to compare the effects of occipital cortex stimulation.

#### Occipital cortex vs Cz

**Figure 5:**
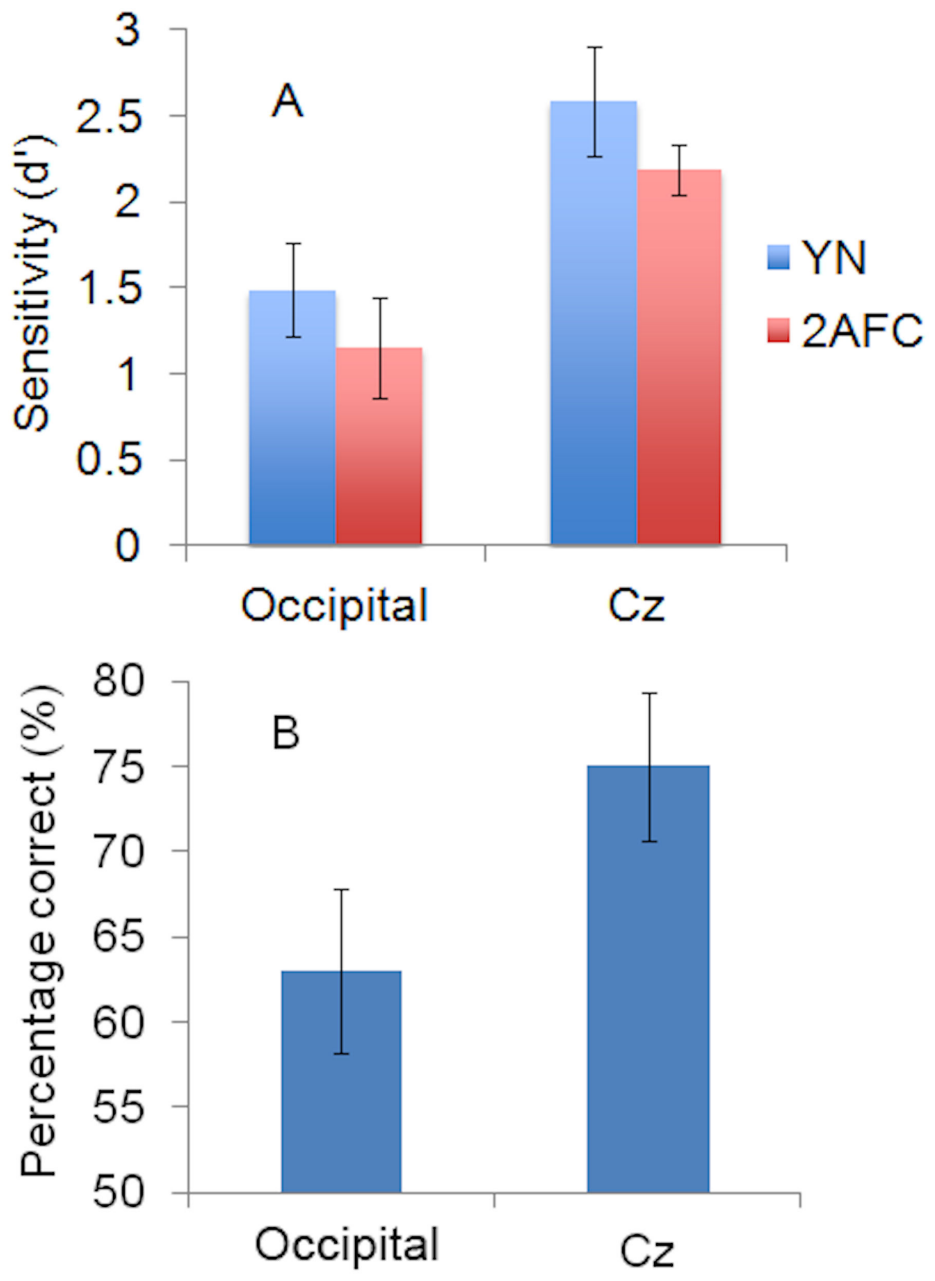
Evidence for TMS-induced Unconscious Vision. **A**. Visual sensitivity under Yes-No (YN) and Forced-Choice (FC) response conditions during TMS stimulation of Occipital Cortex and Cz. **B**. Percentage of correct orientation judgments on miss trials during TMS to Occipital Cortex and Cz. Vertical bars show the standard error of the mean.

The 2x2 repeated measures ANOVA indicated a significant main effect of condition (occipital cortex vs Cz), such that visual sensitivity (d') was again significantly lower under stimulation of occipital cortex (d' = 1.32, SE = .28) than under that of Cz (d' = 2.39, SE = .24), *F*_1, 9_ = 17.14, *p* = .003. This reiterates that TMS to occipital cortex causes a significant impairment in visual awareness. The second main effect, which tested mean sensitivity as a function of psychophysical procedure (YN vs FC), was not significant (d'_YN_ = 2.04, SE = .30, d'_FC_ = 1.67, SE = .22, F_1, 9_ = 4.08, *p* = .07). This suggests that visual performance does not differ between the YN and FC tests independently of other factors. The ANOVA analysis also indicated that during occipital cortex stimulation, sensitivity in the FC task was less than sensitivity in the YN task (mean difference = .34, SE = .15). However, this was not significantly different from the corresponding comparison in the Cz condition (mean difference = .40, SE = .26), *F*_1, 9_ = .10, *p* = .77. The absence of interaction between TMS site and visual sensitivity during the YN and FC tasks, illustrated in Figure 5A, points out that TMS to occipital cortex did not cause a change in visual functioning that could be interpreted as unconscious vision. Nevertheless, percent correct performance on the FC task was, on average, above-chance on trials where subjects reported no awareness of the orientation of the visual stimulus. As shown in Figure 5B, FC accuracy on miss trials was above-chance in both the occipital cortex condition (M = 63%, SE = .05) and Cz condition (M = 75%, SE = .04). Compared to chance (*t* test-value = 50%), reportedly unconscious FC performance was significant in both the occipital cortex condition (*t*_9_ = 2.61, *p* = .03) and Cz condition (*t*_9_ = 5.91, *p* = .0001) and evidence of this phenomenon did not differ significantly between the conditions (SE = .08, *t*_9_ = 1.56, *p* = .15).

### Discussion

In Experiment 2, subjects were again above-chance when forced to discriminate the orientation of visual stimuli that were presented within the TMS-affected area of their visual field, even when they reported no awareness of the orientation of these visual stimuli under YN procedures. Furthermore, the amount by which FC accuracy exceeded chance performance in the absence of awareness was greater in this experiment than in the previous one. However, when sensitivity was indexed as a bias-free measure, d', FC visual performance was not greater than YN visual performance. There was also no difference in the degree of evidence for unconscious vision between the occipital cortex and Cz (control) conditions. This further confirms that above-chance FC accuracy in the absence of reported awareness was not due to unconscious vision. Rather, the results verify that the use of high response criteria under YN procedures influenced visual performance. These criteria meant that subjects tended to report their visual awareness in a conservative manner, in turn retaining visual capacity during the reported absence of awareness. These findings should be considered in light of what previous researchers have called TMS-induced “type 2” blindsight [11]. 

## Experiment 3

Experiment 3 differed from Experiment 2 in two respects. In Experiment 3, subjects made the FC orientation judgment using a grasping gesture, which demonstrated how they would pick up the stimulus if it were physically present on the computer monitor. This is in contrast to Experiments 1 and 2, in which subjects made the FC response by pressing keys labeled as left and right. The second change that was made regarded the orientation of the stimulus. In order to avoid ambiguity in the grasping gesture, the visual stimulus was changed from a bar that was left vs right of vertical to a bar that was horizontal vs vertical. It was thought that the FC grasping gesture would more effectively capture unconscious visual processing than the key-press responses used in the previous two experiments. This was based on evidence that has linked unconscious visual processing to automatic motor behaviours through the activity of the parietal cortex in the dorsal visual stream [29]. For example, Christensen et al. [12] found evidence of “action blindsight”, whereby corrective reaching movements were made in response to visual stimuli that TMS to occipital cortex had suppressed from subjects’ conscious awareness. Based on this research, it was hypothesised that, in contrast to the previous experiments, TMS-induced unconscious vision would arise from true differences in visual sensitivity between the YN (verbal) and FC (action) tasks during stimulation of occipital cortex, but not during stimulation of Cz. Note that Experiment 3 was the same as Experiment 2 in that subjects reported their awareness of the orientation of the visual stimulus and not only its presence.

### Method

#### Subjects

Ten (5 female; mean age 20; age range, 19 to 40 years) first-year psychology undergraduate students participated in this Experiment in exchange for course credit. They were neurologically healthy and had normal or corrected-to-normal vision. All subjects gave informed consent and completed a TMS safety-screening questionnaire prior to completing the experiment, which was approved by the Human Research Ethics Committee at the University of Sydney. All other aspects of the methodology in Experiment 3, aside from those mentioned in the Introduction, were identical to Experiments 1 and 2.

#### Phosphene location

 Phosphenes (and thus visual stimuli) were located in the lower right quadrant of the visual field for 5 subjects and in the lower left quadrant of the visual field for 5 other subjects. Phosphenes did not occur exclusively in the upper half of the visual field, but did extend into this area from the lower right quadrant for 2 subjects.

### Results

#### Cz vs no TMS

Sensitivity in the YN task was not significantly different, on average, between the Cz condition (d' = 2.02, SE = .29) and no TMS condition (d' = 1.81, SE = .27), *t*_9_ = - 1. 81, *p* = .10, nor was sensitivity in the FC task significantly different between these two conditions (d'_Cz_ = 1.71, SE = .20; d'_no TMS_ = 1.61, SD = .18), *t*_9_ = - .95, *p* = .37. This finding confirms that extraneous TMS factors did not have notable effect on visual performance.

#### Occipital cortex vs Cz

**Figure 6:**
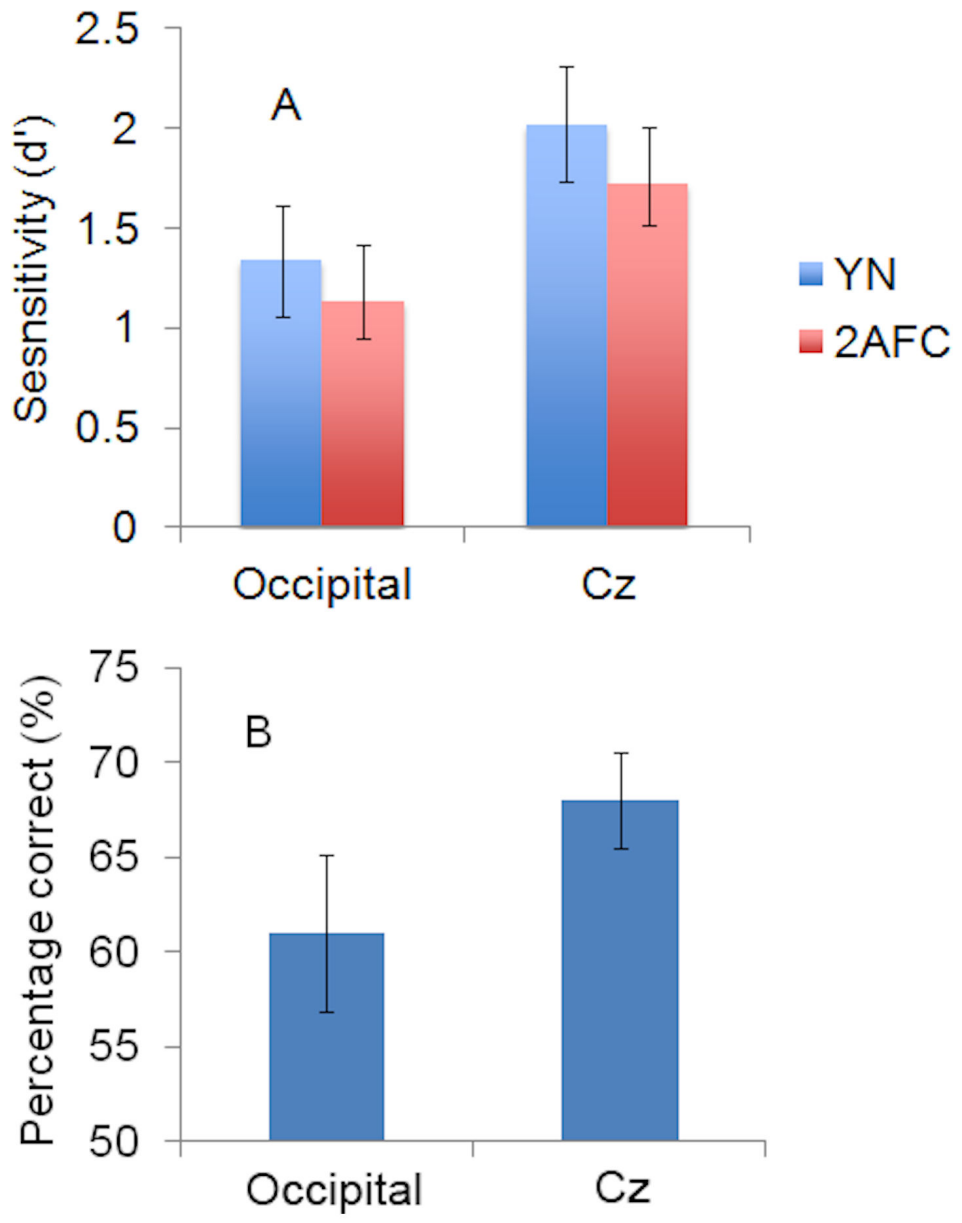
Evidence for TMS-induced Unconscious Vision. **A**. Visual sensitivity under Yes-No (YN) and Forced-Choice (FC) response conditions during TMS stimulation of Occipital Cortex and Cz. **B**. Percentage of correct orientation judgments on miss trials during TMS to Occipital Cortex and Cz. Vertical bars show the standard error of the mean.

The 2x2 repeated measures ANOVA again indicated a significant main effect of condition (occipital cortex vs Cz), such that visual sensitivity (d') was significantly lower under stimulation of occipital cortex (d' = 1.24, SE = .24) than under that of Cz (d' = 1.87, SE = .25), *F*_1, 9_ = 18.36, *p* = .002. This reconfirms that TMS to occipital cortex led to impaired visual awareness. The main effect of test (YN vs FC) was again not significant (d'_YN_ = 1.68, SE = .29, d'_FC_ = 1.43, SE = .20, F_1, 9_ = 1.526, *p* = .248), reiterating that the YN and FC tests are equivalent indices of visual performance independently of other factors. During occipital cortex stimulation, sensitivity in the FC task was less than sensitivity in the YN task (mean difference = .20, SE = .18) and this was not significantly different from the corresponding comparison in the Cz condition (mean difference = .30, SE = .25), *F*_(1, 9)_ = .42, *p* = .53. The absence of interaction between the site of TMS stimulation and visual sensitivity in the YN and FC tasks, illustrated in Figure 6A, points out that TMS to occipital cortex did not cause a change in visual functioning that could be interpreted as unconscious vision. Nevertheless, using a measure that is not independent of bias (percentage of correct responses), evidence for TMS-induced unconscious vision was obtained. Percent correct performance on the FC task was, on average, above-chance on trials where subjects reported no awareness of the visual stimulus. FC accuracy on miss trials was significantly above-chance in both the occipital cortex condition (M = 61%, SE = .03, *t*_9_ = 4.37, *p* = .002) and Cz condition (mean = 68%, SE = .04, *t*_9_ = 4.36, *p* = .002), as shown in Figure 6B. Evidence of unconscious vision was not significantly different between the occipital cortex and Cz conditions (SE = .04, *t*_9_ = 1.81, *p* = .10).

### Discussion

As in the previous two experiments, subjects were above-chance when making forced responses to the orientation of a bar even though they had reported that they were not aware of its orientation. Nevertheless, above-chance FC accuracy in the reported absence of awareness was not underpinned by unconscious visual processing, given bias-free measures of visual performance (d') failed to show a significant difference between the YN and FC tasks. This was in spite of the fact that a motoric FC response was specifically chosen in Experiment 3 to assess unconscious processing in the dorsal visual stream. The findings of Experiment 3 reiterate those of Experiments 1 and 2 in showing that a high decision criterion in the YN task underestimates the respondent’s actual level of visual awareness. In turn, evidence for TMS-induced unconscious vision is an artifact of a decision criterion in the YN judgment. 

## General Discussion

The application of TMS to occipital cortex in normal subjects induced a temporary loss of function in this area, which led to an impairment in the corresponding region of the visual field. Subjects were nonetheless above-chance when forced to make specific judgments about reportedly unseen visual stimuli presented within the TMS-affected region. As in previous studies, this finding suggested that there were changes in visuocortical functioning following loss of function of occipital cortex such that visual sensitivity was greater under forced-choice (FC) conditions than under those of the Yes-No (YN) task. This is typically explained in terms of unconscious visual processing. The purpose of this study was to address the argument that extant cases of TMS-induced unconscious vision are not instances of unconscious vision, but result from the use of a response criterion under YN measures of awareness. This issue was addressed by verifying the effects of occipital TMS on vision using d', which is independent of response criteria. This analysis revealed that, during the TMS-induced loss of function of occipital cortex, sensitivity was not greater for the FC response than for the YN judgment. This result was highly consistent across three different experiments, which used several distinct response procedures in order to maximize the chance that potentially unconscious visual processing would be captured. The logical conclusion of the present study is that TMS-induced unconscious vision, operationally defined as above-chance FC accuracy in the reported absence of awareness, is not actually a phenomenon of unconscious visual experience: instead it may be reasonably attributed to an artifact of the use of a response criterion for YN measures of awareness but not for FC responses. 

A notable increase in evidence of unconscious vision was found to occur in Experiments 2 and 3, in comparison to Experiment 1. This finding may relate to the fact that first-year university undergraduates were tested in the second and third experiments, whereas individuals with experience in psychophysical testing procedures participated in the first experiment. This suggests that experience with perceptual tasks and the ability to monitor response behaviour are important factors influencing response bias. Alternatively, the difference in evidence for unconscious vision might be attributed to the fact that subjects in Experiment 1 had to respond yes or no to whether they saw a stimulus, whereas subjects in Experiments 2 and 3 responded yes or no to whether they saw its orientation. The latter judgement may well encourage subjects to use a higher response criterion because it requires them to report on a more specific property of the stimulus. As already noted, anything that elevates the subjects’ response criterion in the YN task will increase the miss rate and thus increase the accuracy of FC responses on those missed trials.

Regardless of the factors influencing the decision criterion, the present findings highlight a fundamental issue in the research of unconscious vision: the use of dichotomous measures of visual awareness (see Overgaard [30] for a review of this and several other issues concerning blindsight). These measures, such as the YN task, rely on the use of subjective response criteria, which allows these tests to distort the measurement of visual awareness. Such distortion was particularly evident in Experiment 2, in which subjects were able to correctly discriminate up to 75% of “unseen” visual stimuli, even in the absence of disruption to occipital cortex. (i.e. TMS to the control site, Cz). Nevertheless, objective levels of sensitivity to the visual stimulus (d') did reveal that subjects may have underestimated their visual awareness during the experiment. Overgaard et al. [19] similarly found that blindsight patient GR would typically report having no conscious awareness of visual stimuli seen as an “almost clear image” or “weak glimpse”. This meant that when GR was truly unaware of any visual stimulus, indicated by use of the lowest level on the Perceptual Awareness Scale (PAS), labelled “nothing seen”, evidence of blindsight was not found. As in the present study, neurologically normal subjects (with intact occipital functioning) were very similar: during TMS to occipital cortex, response patterns were consistent with TMS-induced unconscious vision only in association with visual stimuli rated as a “weak glimpse” and “almost clear image” under the PAS [31]. Evidence therefore clearly demonstrates that dichotomous measures, such as the YN, commonly distort the measurement of visual awareness. In turn, this distortion has clear potential to produce spurious evidence of unconscious vision.

The present findings raise doubt about the validity of previous instances of TMS-induced unconscious vision. To the authors’ knowledge, Ro et al. [14] was the first study to this phenomenon, which they refer to as TMS-induced blindsight. In this study, subjects were able to localise visual stimuli using saccadic eye movements, despite reporting that they did not see these stimuli following TMS stimulation of occipital cortex. A follow-up study by Boyer et al. [11] also found evidence for unconscious visual processing of colour and orientation in the TMS-induced absence of visual awareness. Several other similar studies have since been conducted, leading to claims of TMS-induced “action blindsight” [12] and “affective blindsight” [32]. In addition to the fact that these studies have not conducted the necessary signal detection analysis of subjects’ responses, it is concerning that control stimulation data (e.g. Cz or no TMS) are rarely reported. The current study found clear evidence that reportedly unconscious vision caused by occipital TMS was not actually unconscious, not only through the use of a bias-free measure of visual performance (d'), but also in the fact that equivalent evidence for unconscious vision occurred under control conditions, in which occipital cortex functioning was not disrupted. It is therefore highly important that future studies carry out signal detection analysis on psychophysical data and use appropriate controls against which to compare the effects of TMS on visual cortex. 

Despite the quite different effects of TMS to occipital cortex compared to permanent lesions of this area, it is nonetheless pertinent to discuss the current findings in relation to clinical blindsight. In past experiments, such as Azzopardi and Cowey [2], SDT has confirmed that in some individuals who have suffered permanent loss of function of V1, such as GY, there is a true difference in visual sensitivity (d') between the YN and FC tasks, such that it is significantly greater in the latter compared to the former. It has been suggested that GY’s permanent injury to V1 may have had “unusual” effects on his visuocortical processing [3]. It may be, for example, that following lesions of V1 there are neuroplastic changes that lead to the activation of subcortical visual pathways as the brain attempts to restore visual functioning [30]. In support of this possibility, some evidence suggests that dorsal areas, such as the middle temporal area, are more strongly activated in response to visual stimuli in de-striated individuals than are ventral visual areas [33,34]. In turn, if dorsal areas are more strongly activated by the FC response procedure than by that of the YN task, this would account for the discrepancy in GY’s visual sensitivity between these two kinds of task.

Nevertheless, other experiments examining different blindsight patients are few, and a number of more recent studies have found evidence contrary to the belief that so-called blindsight is actually unconscious. In an interview with Persaud and Lau [35], for example, GY reported that he does have conscious visual experiences when stimuli are presented in his defective visual region. Specifically, GY reported having “qualia”, or inner qualitative experiences [36], which were more apparent when the brightness of stimuli was increased - consistent with conscious near-threshold vision. Similarly, GY, and another blindsight patient, MS, were only above-chance when discriminating stimuli within their defective visual regions when these stimuli had sharp spatial boundaries [37], which is again at odds with the premise of an unconscious nature of the phenomenon. Continuing research also suggests that most, if not all, extant cases of blindsight are “type 2”, a subtype in which patients report having vague sensory experiences, such as “moving waves” [38] and “dark shadows” [39] within their scotomata. By contrast, there is little evidence that individuals with lesions of striate cortex can perform visual tasks at above-chance levels in the complete absence of visual awareness (“type 1” blindsight), as Overgaard [19] demonstrated with the graded PAS measure administered to blindsight patient GR. This line of evidence tentatively suggests that there may be some similarity between clinical and TMS-induced instances of unconscious vision as it relates to the phenomenon of blindsight, and that in both cases vision may be more suitably described as degraded, rather than unconscious.

It would be informative if future research used the methodology employed here to examine TMS-induced unconscious vision for different visual stimuli. This study employed a visual stimulus that varied in its orientation, but previous studies have examined stimuli that vary in colour, facial expression, and motion. It is conceivable, for example, that an attribute such as facial expression might have stronger subcortical underpinnings than other stimulus properties due to its evolutionary salience. Along the same lines, the automatic localization of visual stimuli through saccadic eye movements, which has been associated with activation of the superior colliculus, may also have more active subcortical underpinnings than other stimulus properties.

## Conclusions

This study has provided strong convergent evidence that TMS-induced above-chance FC accuracy in the reported absence of visual awareness in normal individuals is not attributable to genuine differences in visual sensitivity between YN and FC measures of vision. Rather, the effect of a response criterion on the YN judgment makes it appear as though visual stimuli are processed in the absence of awareness. This finding highlights the fact that the dichotomous YN procedure is not independent of subjectivity, and for this reason can and does lead to marked distortion in the measurement of visual awareness.
